# Association of serum creatinine with hepatic steatosis and fibrosis: a cross-sectional study

**DOI:** 10.1186/s12876-022-02437-0

**Published:** 2022-07-27

**Authors:** Juan Ma, Zhongcao Wei, Qian Wang, Xiaolan Lu, Zhihua Zhou, Ruohan Li, Qiuai Shu, Yixin Liu, Jinhai Wang, Na Liu, Haitao Shi

**Affiliations:** 1 Department of Gastroenterology, The Second Affiliated Hospital of Xi’an Jiaotong University, NO.157 Xiwu Road, Xi’an, 710004 Shaanxi China; 2 Health Management Department, The Second Affiliated Hospital of Xi’an Jiaotong University, NO.157 Xiwu Road, Xi’an, 710004 Shaanxi China; 3 Department of Gastroenterology, Shanghai Pudong Hospital, Fudan University, 2800 Gongwei Road, Huinan Town, Pudong, Shanghai, 201399 China; 4 Department of Critical Care Medicine, The Second Affiliated Hospital of Xi’an Jiaotong University, NO.157 Xiwu Road, Xi’an, 710004 Shaanxi China

**Keywords:** Metabolic syndrome, Hepatic steatosis, Serum creatinine, Hepatic fibrosis

## Abstract

**Background:**

Recent studies have shown that chronic kidney disease (CKD) prevalence is significantly higher in patients with hepatic steatosis (HS); however, it remains unclear whether HS is associated with serum creatinine (SCr). We aimed to explore the association between SCr levels and HS in a Chinese population.

**Methods:**

We performed a cross-sectional study among 56,569 Chinese individuals. SCr level, other clinical and laboratory parameters, abdominal ultrasound and noninvasive fibrosis scores were extracted, and the fibrosis 4 score (FIB-4) was calculated.

**Results:**

A total of 27.1% of the subjects had HS. After 1:1 propensity score matching (PSM) according to sex and age, we included 13,301 subjects with HS and 13,301 subjects without HS. SCr levels were significantly higher in the HS group than in the non-HS group [73.19 ± 15.14(μmoI/L) vs. 71.75 ± 17.49(μmoI/L), *p* < 0.001]. Univariate and multivariate regression analyses showed a positive association between SCr and the prevalence of HS. Stepwise regression analysis showed that the association between SCr and HS was independent of other metabolic syndrome components. The prevalence of HS increased significantly with increasing SCr levels. Metabolism-related indicators and liver enzymes were significantly higher in the HS group than in the non-HS group; furthermore, these parameters increased with increasing SCr levels. FIB-4 was significantly higher in the HS group than in the non-HS group but did not show an increasing trend with increasing SCr levels.

**Conclusions:**

Our results showed an independent association between SCr level and HS risk in a Chinese population.

## Introduction

Hepatic steatosis (HS) is defined as the intracellular accumulation of triglycerides in more than 5% of hepatocytes [1]. HS is the most common chronic liver disease, and its incidence has been increasing in recent years [2]. HS has been shown to be strongly associated with obesity, insulin resistance, inflammation, and even cardiovascular disease (CVD) and chronic kidney disease (CKD) [3–9]. While HS can be benign, it is often associated with the development of inflammation and progression to fibrosis and cirrhosis. Recent studies have shown that at least a quarter of patients with HS develop fibrosis within 6 years, and their mortality rates increase by ~ 10% within 20 years, primarily due to cancer and cirrhosis [10, 11]. Recently, extrahepatic complications have attracted increasing attention as risk factors for diabetes mellitus, atherosclerosis and metabolic syndrome. In particular, the role of HS in the pathogenesis of CKD has attracted widespread attention [12–14]. Studies have shown an increased prevalence and incidence of CKD in patients with HS [15].

Serum creatinine (SCr), a biosynthetic product of phosphocreatine metabolism in muscle, is widely used in clinical practice to assess renal function and is more direct and readily available than glomerular filtration rate (GFR). Several clinical studies have shown that even within the normal range, higher SCr is strongly associated with the risk of several diseases, such as cardiovascular disease and diabetes [16, 17]. Currently, few clinical studies have investigated the relationship between HS and SCr levels and HS-accompanied fibrosis. The relationship between SCr levels in individuals with HS and HS-accompanied fibrosis is still uncertain. Understanding this aspect may lead to further interpretation of the underlying mechanisms of HS and HS-accompanied fibrosis and may be of clinical importance for prevention. We performed the first cross-sectional study to investigate the changes in SCr levels in a large Chinese population of subjects with ultrasound-confirmed HS and HS-accompanied fibrosis.

## Patients and methods

### Study population

**Fig. 1:**
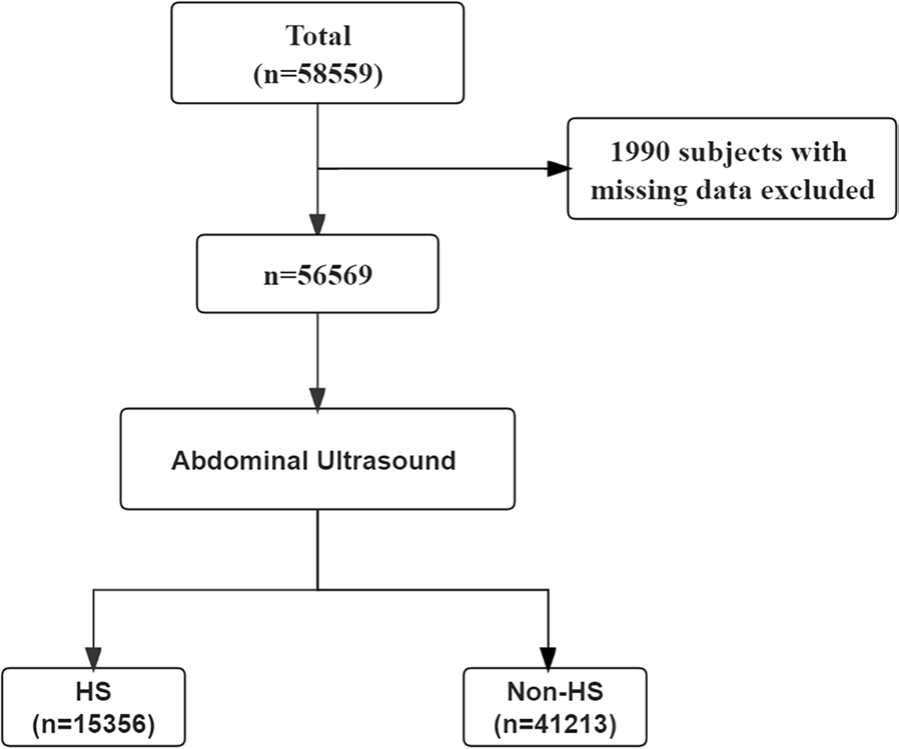
Flow chart for data inclusion. HS, Hepatic steatosis; Non-HS, Non-Hepatic steatosis

The study cohort consisted of 56,569 individuals who voluntarily visited the Health Promotion Centre of Second Affiliated Hospital of Xi’an Jiaotong University for regular health examinations between January and December 2020. The Second Affiliated Hospital of Xi’an Jiaotong University, a large general hospital located in northwest China, has a wide coverage of medical checkups, and the attended population extends throughout the northwest. Individuals with complete data on age, sex, body mass index (BMI), fasting blood glucose (FBG), systolic blood pressure (SBP), diastolic blood pressure (DBP), SCr, triglycerides (TGs), high-density lipoprotein cholesterol (HDL-c), low-density lipoprotein cholesterol (LDL-c), total cholesterol (TC), alanine aminotransferase (ALT) and aspartate aminotransferase (AST) were included in the analysis, and the flow chart is shown in Fig. 1.

A total of 56,569 subjects were eventually included in this study. The final included subjects were divided into HS and non-HS groups. There were 15,356 individuals with HS and 41,213 non-HS participants. To balance the confounding factors, we performed 1:1 propensity score matching (PSM) using sex and age with a caliper of 0.001. The study was approved by the Ethics Committee of Second Affiliated Hospital of Xi’an Jiaotong University. Given the retrospective nature of the study, we were granted a waiver of informed consent. All patients are anonymous and their information is not public.

### Clinical and experimental data collection

Venous blood was obtained from all participants in the morning after overnight fasting, and serum was isolated for biochemical analysis without refrigeration. The variables analyzed included ALT, AST, FBG, TC, TGs, HDL-c, LDL-c, FBG and SCr. Abdominal ultrasound was performed by two experienced technicians on an EUB-6500 Hitachi ultrasound machine (Hitachi, Tokyo, Japan).

### Definitions

HS was diagnosed by liver ultrasonography performed on all subjects by expert physicians using the EUB-6500 Hitachi ultrasound machine, who were unaware of the study objectives and laboratory results. diagnostic indicators of HS included liver brightness, hepatorenal contrast, intrahepatic vascularity, ultrasound presentation of the liver parenchyma and diaphragm. All participants underwent a physical examination at the Health Screening Center of the Second Affiliated Hospital of Xi’an Jiaotong University. BMI was defined as weight in kilograms divided by height in meters squared (kg/m^2^). SBP and DBP were measured by the same sphygmomanometer. Hepatic steatosis was defined using abdominal ultrasound. The severity of liver fibrosis was assessed noninvasively by the FIB-4 score (FIB-4 = age × AST (IU/L)/[platelet count (× 109/L) × ALT (IU/L)^1/2^]). The lower and upper limits of FIB-4 were 1.3 and 2.67, respectively [18].

GFR is the primary function of the kidney and is measured to assess overall health status. The Chronic Kidney Disease Epidemiology Collaborative (CKD-EPI) equation is based on the creatinine replacement formula[19]. The equation is expressed as: eGFR = 141 × [the minimum of standardized SCr (mg/dL)/κ or 1]^α^ × [the maximum of standardized SCr (mg/dL)/κ or 1] ^−1.209^ × 0.993^age^ × (1.018 if female) × (1.159 if black), where κ is 0.7 for females and 0.9 for males and α is − 0.329 for females and − 0.411 for males.

### Statistical analysis

To balance the confounding factors, we performed propensity score matching (PSM) using a logistic regression model for the following variables: sex and age. The two groups were matched 1:1 with a caliper of 0.001.

Normally distributed data are expressed as the means ± standard deviations (SDs). Categorical variables are expressed as frequencies and percentages. Continuous variables were compared using Student’s t test, and categorical variables were compared using the χ^2^ test. Comparisons between multiple groups were made using ANOVA. The metabolism-related parameters, liver enzyme profiles, and FIB-4 scores of the study subjects were compared between SCr quartiles. We explored the correlates of HS through univariate and multivariate logistic regression analysis; that is, significant factors (*p* < 0.05) were included in the univariate regression and further validated in the multivariate regression. Covariables included sex, age, systolic blood pressure, diastolic blood pressure, lipid profile, and liver enzyme profile. All tests were two-tailed, and results *p* < 0.05 were considered statistically significant. Statistical analyses were performed with SPSS software version 23.0 for Windows (SPSS Inc., Chicago, IL, USA).

## Results

### Characteristics of the study subjects

A total of 56,569 subjects were ultimately included in this study. The demographic and clinical characteristics of the pre- and post-PSM study populations are shown in Table 1. Before PSM, there were 41,213 participants in the non-HS group and 15,356 participants in the HS group, with a rate of HS prevalence of 27.1%. Before PSM, age and sex showed significant differences between the two groups. However, these indicators became similar after PSM. Before PSM, compared with the non-HS group, BMI, SBP, DBP, FBG, TC, LDL-c, ALT, AST and FIB-4 levels were significantly increased (all *p* < 0.05), while TGs and HDL-c were not significantly different in HS subjects. This statistical regularity was also maintained between the two groups after PSM. As expected, after PSM, the SCr level in the HS group was significantly higher than that in the non-HS group [73.19 ± 15.14(μmoI/L) vs. 71.75 ± 17.49(μmoI/L), *p* < 0.001, respectively].

**Table 1:** Demographic and biochemical characteristics before and after Propensity Score Matching (PSM)

Variables	Before PSM		*p*	After PSM			*p*
	Non-HS (n = 41,213)	HS (n = 15,356)		Non-HS (n = 13,301)	HS (n = 13,301)	OR (95% CI)	
Male (%)	23,613(57.3)	8153(53.1)	< 0.0001	7465(56.1)	7417(55.8)	0.985 (0.938–1.034)	0.540
Age (years)	41.64 ± 14.30	43.03 ± 14.10	< 0.0001	43.49 ± 14.71	43.45 ± 14.33	1.000 (0.998–1.010)	0.830
BMI (kg/m^2^)	23.86 ± 3.45	24.10 ± 3.44	< 0.0001	23.69 ± 3.43	24.10 ± 3.57	1.034 (1.027–1.042)	< 0.0001
SBP (mmHg)	122.39 ± 15.68	123.54 ± 16.11	< 0.0001	121.54 ± 16.16	123.62 ± 16.07	1.008 (1.007–1.010)	< 0.001
DBP (mmHg)	78.52 ± 10.52	79.27 ± 11.10	< 0.0001	78.54 ± 10.83	79.26 ± 11.07	1.006 (1.004–1.008)	< 0.0001
FBG (mmol/L)	5.34 ± 1.19	5.60 ± 1.16	< 0.0001	5.30 ± 1.19	5.61 ± 1.13	1.319 (1.284–1.355)	< 0.0001
TC (mmol/L)	4.36 ± 0.87	4.43 ± 0.84	< 0.0001	4.31 ± 0.85	4.44 ± 0.84	1.214 (1.179–1.251)	< 0.0001
TG (mmol/L)	1.51 ± 1.21	1.54 ± 1.23	0.110	1.55 ± 1.24	1.56 ± 1.16	1.010 (0.990–1.030)	0.352
HDL-c (mmol/L)	1.25 ± 0.03	1.25 ± 0.31	0.680	1.23 ± 0.29	1.24 ± 0.30	1.073 (0.989–1.165)	0.088
LDL-c (mmol/L)	2.63 ± 0.74	2.65 ± 0.75	0.018	2.60 ± 0.70	2.66 ± 0.73	1.133 (1.096–1.172)	0.001
ALT (U/L)	23.40 ± 19.75	24.39 ± 18.15	< 0.0001	22.24 ± 18.08	24.38 ± 18.36	1.027 (1.023–1.030)	< 0.0001
AST (U/L)	22.64 ± 10.08	23.21 ± 8.75	< 0.0001	22.36 ± 9.83	24.23 ± 8.86	1.007 (1.005–1.008)	< 0.0001
SCr (μmol/L)	71.51 ± 18.21	73.18 ± 15.46	< 0.0001	71.75 ± 17.49	73.19 ± 15.14	1.006 (1.005–1.008)	< 0.0001
eGFR [mL/(min*1.73 m^2^)]	125.50 ± 55.56	123.77 ± 51.84	0.001	102.45 ± 15.66	100.97 ± 15.39	0.995(0.994–0.995)	< 0.0001
FIB-4	1.03 ± 0.68	1.05 ± 0.73	< 0.0001	1.10 ± 0.71	1.14 ± 0.76	1.064 (1.029–1.100)	< 0.0001

Data are presented as the means ± standard deviations (SDs), number (percentage). ALT, alanine aminotransferase; AST, aspartate aminotransferase; BMI, body mass index; CI, confidence interval; DBP, diastolic blood pressure; FBG, fasting blood glucose; FIB4, fibrosis 4 score; HDL-c, high-density lipoprotein cholesterol; HS, Hepatic steatosis; LDL-c, low-density lipoprotein cholesterol; OR, odds ratio; SBP, systolic blood pressure; SCr, serum creatinine; TC, total cholesterol; TG, triglycerides; eGFR, Glomerular filtration rate

* denotes the multiplicative relationship

### Relevant factors for the prevalence of HS

**Fig. 2:**
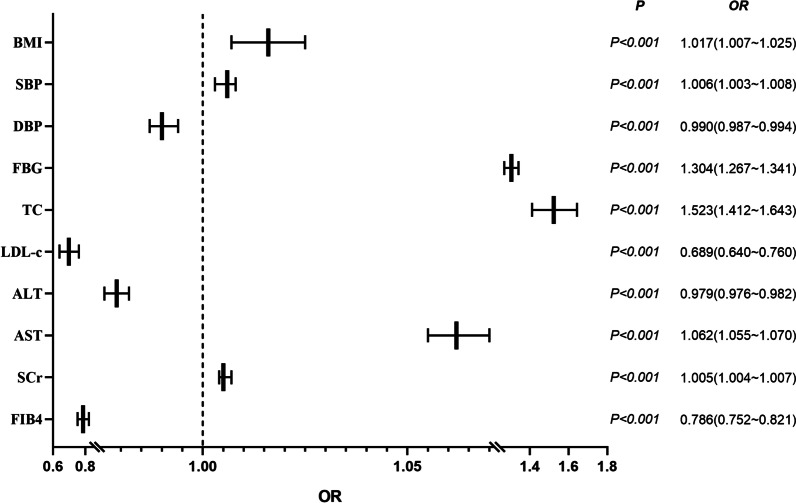
Forest diagram of Relevant factors for the incidence of hepatic steatosis (HS). HS, Hepatic steatosis; OR, odds ratio; CI, confidence interval. BMI, body mass index; SBP, systolic blood pressure; DBP, diastolic blood pressure; TC, total cholesterol; TG, triglycerides; HDL-c, high-density lipoprotein cholesterol; LDL-c, low-density lipoprotein cholesterol; ALT, alanine aminotransferase; AST, aspartate aminotransferase; SCr, serum creatinine; FBG, fasting blood glucose. FIB4, fibrosis 4 score

In the univariate analysis, factors associated with HS prevalence included BMI, SBP, DBP, FBG, TC, LDL-c, ALT, AST, SCr, and FIB-4, as shown in Table 1. To investigate the independent correlates of HS prevalence, after PSM, we further used multivariate logistic regression analysis to determine the independent determinants of HS, including BMI (odds ratio [OR]: 1.017, 95% confidence intervals [CI]: 1.007 ~ 1.025, *p* < 0.05), FBG (OR: 1.304, 95% CI: 1.267 ~ 1.341, *p* < 0.001), TC (OR: 1.523, 95% CI: 1.421 ~ 1.643, *p* < 0.001), LDL-c (OR: 0.689, 95% CI: 0.640 ~ 0.760, *p* < 0.05), SCr (OR: 1.005, 95% CI: 1.004 ~ 1.007, *p* < 0.001), AST (OR: 1.062, 95% CI: 1.055 ~ 1.070, *p* < 0.001), ALT (OR: 0.979, 95% CI: 0.976 ~ 0.982, *p* < 0.001) and FIB-4 (OR: 0.786, 95% CI: 0.752 ~ 0.821, *p* < 0.001), as shown in Fig. 2.

### Logistic regression analysis for the association of SCr with HS

As shown in Table 2, multiple logistic analysis indicated SCr was positively associated with the prevalence of HS (OR: 1.012, 95% CI: 1.010–1.014, *p* < 0.001) after adjustment for age and sex (Model 1). After further adjustment for BMI, SBP, DBP, TGs, TC, HDL-c, LDL-c, and FBG, the association between Scr and HS remained significant (OR: 1.010, 95% CI: 1.008–1.013, *p* < 0.001, Model 2). In addition, SCr still showed a significant association with HS after additional adjustment for ALT, AST and FIB-4 (OR: 1.010, 95% CI: 1.007–1.012, *p* < 0.001, Model 3). According to the CKD-EPI_SCr_ equation, the eGFR was significantly decreased in HS group compared to non-HS group.

**Table 2:** Logistic regression analysis for the association of serum creatinine (SCr) with hepatic steatosis (HS)

SCr	OR (95%CI)	*P*
Model 1	1.012 (1.010–1.014)	< 0.001
Model 2	1.010 (1.008–1.013)	< 0.001
Model 3	1.010 (1.007–1.012)	< 0.001

Model 1 was adjusted for age and gender. Model 2 was adjusted for the covariates of model 1 plus body mass index, systolic blood pressure, diastolic blood pressure triglycerides, high-density lipoprotein cholesterol, low-density lipoprotein cholesterol, total cholesterol, and Fasting blood glucose. Model 3 was adjusted for the covariates of model 2 plus alanine transaminase, aspartate transaminase and fibrosis 4 score. Odds ratios and 95% CIs were calculated per 1-SD increment of SCr. CI, confidence interval; OR, odds ratio; SCr, serum creatinine

### HS increases with progressively higher SCr

**Fig. 3:**
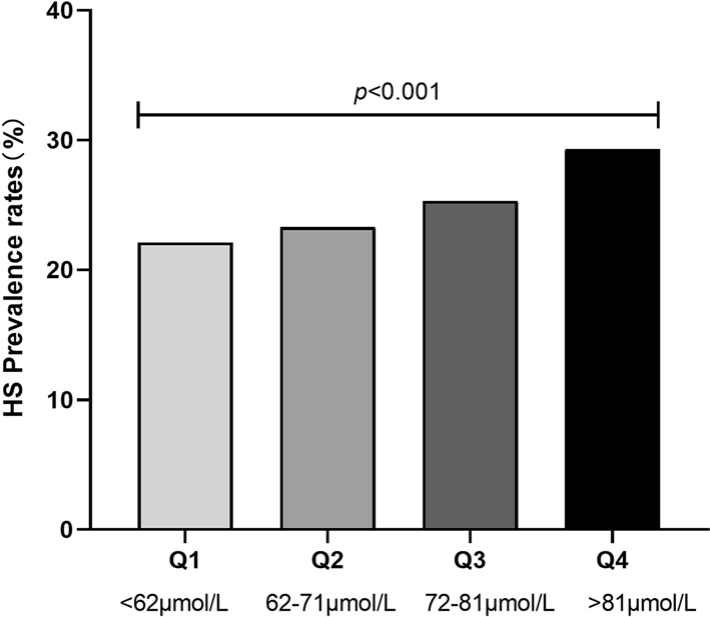
Relationship between serum serum creatinine (SCr) and the prevalence of hepatic steatosis (HS)

To investigate the relationship between SCr and the prevalence of HS, we divided the subjects into quartiles (Q) according to baseline SCr (Q1 < 62 μmol/L; Q2, 62 ≤ Q2 ≤ 71 μmol/L; 71 < Q3 ≤ 81 μmol/L; Q4 > 81 μmol/L). As shown in Fig. 3, the prevalence of HS tended to increase with increasing SCr. Compared with subjects in Q1 of SCr (22.1%), the prevalence of HS in subjects in Q2, Q3 and Q4 was 23.3, 25.3 and 29.3%, respectively, with a significant trend (*p* < 0.05).

### Metabolism-related parameters in subjects with different SCr quartiles

Compared to subjects in Q1, subjects in Q2, Q3 and Q4 had higher BMI, FBG, TC, TGs, HDL-c, LDL-c, SBP, and DBP (all *p* < 0.001), as shown in Table 3.

**Table 3:** Metabolism-related parameters in subjects with different serum creatinine (SCr) quartiles

Variables	Q1	Q2	Q3	Q4	*p*
	< 62 μmol/L	62–71 μmol/L	72–81 μmol/L	> 81 μmol/L	
BMI	22.57 ± 3.29	23.55 ± 3.48	24.49 ± 0. 04	24.49 ± 3.29	< 0.001
SPB	118.31 ± 16.18	122.45 ± 16.92	124.27 ± 15.11	125.50 ± 15.27	< 0.001
DBP	75.67 ± 10.31	78.65 ± 11.11	80.40 ± 10.70	81.06 ± 10.88	< 0.001
FBG	5.32 ± 1.20	5.47 ± 1.25	5.53 ± 1.22	5.50 ± 1.22	< 0.001
LDL-C	2.53 ± 0.70	2.61 ± 0.71	2.67 ± 0.73	2.68 ± 0.72	< 0.001
HDL-C	1.36 ± 0.30	1.27 ± 0.30	1.18 ± .28	1.13 ± 0.24	< 0.001
TG	1.30 ± 1.17	1.50 ± 1.23	1.68 ± 1.18	1.75 ± 0.24	< 0.001
TC	4.31 ± 0. 82	4.38 ± 0.82	4.41 ± 0.82	4.40 ± 0. 83	< 0.001

Data are presented as mean ± standard deviation (SDs). BMI, body mass index; DBP, diastolic blood pressure; FBG, fasting blood glucose; HDL-c, high-density lipoprotein cholesterol; LDL-c, low-density lipoprotein cholesterol; SBP, systolic blood pressure; TC, total cholesterol; TG, triglycerides

### Association of SCr with liver enzymes or fibrosis in the HS group

**Fig. 4:**
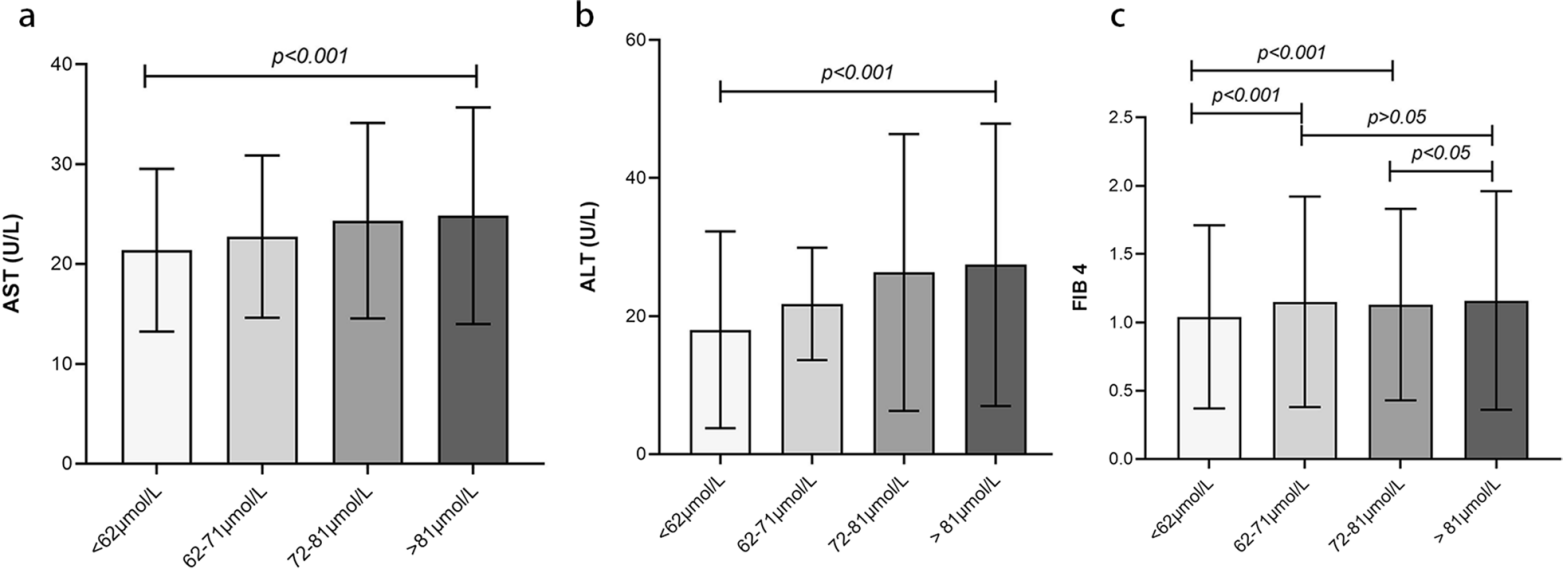
Liver enzyme parameters and fibrosis 4 score (FIB4) of in hepatic steatosis (HS) subjects in different serum creatinine (SCr) quartiles. **a**. Relationship between AST and quartiles of SCr concentration; **b**. Relationship between ALT and quartiles of SCr concentration;**c**. Relationship between FIB4 and quartiles of SCr concentration. AST, aspartate aminotransferase; ALT, alanine aminotransferase; FIB4, fibrosis 4 score

The liver enzyme parameters and noninvasive fibrosis scores in HS subjects in different SCr quartiles are shown in Fig. 4. Subjects in Q2, Q3 and Q4 had higher ALT and AST levels than subjects in Q1 (*p* < 0.001); furthermore, the concentrations of AST and ALT showed an increasing trend with increasing SCr levels. FIB-4 was significantly higher in the HS group than in the non-HS group; however, it did not show an increasing trend with the increasing concentration of SCr.

## Discussion

To the best of our knowledge, this is the first study aimed at evaluating the association between elevated SCr levels and the risk of HS in a large population. In this retrospective study of a large Chinese population, we provide evidence that the risk of HS is positively associated with SCr levels. Further analysis showed that as SCr increased, liver enzyme levels increased, and fibrosis worsened. In addition, SCr level and the prevalence of HS were positively correlated.

HS is the most common chronic liver disease, and the prevalence of HS has been increasing in recent years. In our study, the prevalence of HS was 27.1%, which is consistent with the findings of previous studies [20], however, recent studies have shown that the prevalence of NAFLD and metabolic associated fatty liver disease,(MAFLD) is as high as approximately 35–40% in the contemporary American population[21]. Our results showed that eGFR was significantly decreased in HS patients compared to the non-HS subject population. Several large-scale studies have reported that the prevalence of CKD in patients with HS ranges from 4 to 42% [22–24]. A recent meta-analysis that included 13 studies involving 1,222,032 individuals found that HS was significantly associated with an approximately 1.45-fold increased long-term risk of CKD stage ≥ 3 (HR = 1.43, 95% CI = 1.33–1.54; I^2^ = 60.7%) [12]. In the general US population, liver fibrosis assessed using vibration controlled transient elastography (VCTE) is associated with CKD, specifically with a proteinuric phenotype, but not with traditional risk factors[25]. A study based on 8862 subjects reported that SCr levels were independently associated with HS [26]. Elevated SCr levels, even within the normal range, were associated with a higher risk of NAFLD [26]. Similarly, the current study demonstrates for the first time that elevated SCr levels are an independent risk factor for HS, and logistic regression analysis after adjusting for confounding factors suggests that SCr has a direct effect on HS.

SCr is the most commonly used biomarker of renal function [27]. In the last few years, the role of SCr in cardiovascular disease, type 2 diabetes, and centripetal obesity has been extensively studied [28–30]. Notably, the current analysis suggests that SCr is independently associated with HS. Undoubtedly, the earlier the diagnosis of mild kidney injury in HS, the greater the benefit to the patient, as treatment to reverse this process is most likely to be effective in preventing or slowing disease progression. Our study suggests that SCr is a suitable screening marker for identifying HS risk, in addition to being used for early detection of kidney injury.

The potential mechanisms by which SCr interacts with HS have not been fully elucidated, but several intertwined pathways have been proposed. First, SCr is a commonly used indicator of renal function and can also be used as an indicator of metabolic syndrome (MetS) and its related diseases, with higher SCr levels associated with a higher risk of MetS [31]. Similarly, our findings show that higher SCr levels are strongly associated with MetS components such as FBG, SBP, DBP, TGs and TC. LDL-c (OR: 0.689, 95% CI: 0.640 to 0.760, *p* < 0.05) in a multivariate regression analysis, a finding that we believe may be associated with abnormal liver metabolism. Studies have shown that an increasing number of patients with low LDL-C (< 5%) have been identified in patients with NAFLD[32]. Second, “multiple strikes” is the most widely supported theory for the pathophysiological features of NAFLD [33]. The pathogenesis involves increased circulating free fatty acids with excessive TG accumulation in the liver and insulin resistance (IR), which further promotes inflammatory factor release and endoplasmic reticulum stress. IR, inflammatory and oxidative mediators are commonly involved in the pathogenesis of NAFLD and MetS [34–37]. In addition, our study found that SCr levels were significantly correlated with the liver enzyme profiles of AST, ALT, and ALP, which are markers of liver injury. This finding was independent of IR and other features of MetS. ALT was inverse associated with HS in our finding (OR: 0.979, 95% CI: 0.976 to 0.982, *p* < 0.001). Previous studies have found a linear relationship between ALT and frailty[38], as well as muscle strength[39], However, in NAFLD, the difference between histological severity and ALT elevation was not statistically significant[40]. Therefore, further prospective studies are warranted.

To date, liver biopsy has been considered the gold standard for differentiating the severity of liver disease and estimating the degree of fibrosis [41]. However, liver biopsy is impractical in large epidemiological studies due to the invasive nature of the procedure, complications and poor patient acceptance. FIB-4, a simple index for assessing liver fibrosis, has been shown to reliably predict fibrosis and cirrhosis in NAFLD based on several laboratory tests [42]. Studies have shown that high FIB-4 scores are associated with an increased risk of generalized CKD [43]. In our study, we demonstrated for the first time that FIB-4 scores increased with increasing SCr levels. This finding demonstrates the increase in SCr levels with increasing fibrosis. Although the cutoff value of FIB4 was proposed by Shah et al. [44], according to their findings, the mean value in non-NASH patients was lower than the mean value in the general NAFLD population. Therefore, it is important to be aware of the limitations of noninvasive fibrosis scoring in NAFLD, although the FIB4 index is a simple and inexpensive way to diagnose advanced fibrosis, but its limitations must be considered.

We have to acknowledge several potential limitations in our study. First, we used the FIB-4 score to assess the degree of liver fibrosis rather than liver biopsy. Second, our aim was to study the correlation between HS and SCr, regardless of the cause. This is a limitation of our study because we lack more precise information to determine the etiology of suffering from HS, such as non-alcoholic fatty liver, alcoholic fatty liver disease (AFLD), MAFLD, etc. Therefore, prospective studies are needed to determine whether SCr contributes to the development of HS.

## Conclusions

Our cross-sectional study showed a significant association between SCr and HS. This finding suggests that we should pay more attention to SCr because of its predictive power for HS.

## Data Availability

Data are available upon reasonable request. The datasets used and/or analyzed during the current study are available from the corresponding author on reasonable request.

## Abbreviations

ALT: Alanine aminotransferase
AST: Aspartate aminotransferase
BMI: Body mass index
CI: Confidence interval
DBP: Diastolic blood pressure
FBG: Fasting blood glucose
FIB4: Fibrosis 4 score
HDL-c: High-density lipoprotein cholesterol
HS: Hepatic steatosis
LDL-c: Low-density lipoprotein cholesterol
OR: Odds ratio
PSM: Propensity score matching
SBP: Systolic blood pressure
SCr: Serum creatinine
TC: Total cholesterol
TG: Triglycerides
CKD-EPI: Chronic kidney disease epidemiology collaboration
eGFR: Glomerular filtration rate
NAFLD: Nonalcoholic fatty liver disease
MAFLD: Metabolic associated fatty liver disease
VCTE: Vibration controlled transient elastography
CKD: Chronic kidney disease
CVD: Cardiovascular disease
